# Laparoscopic cholecystectomy in patients with liver cirrhosis: 8 years experience in a tertiary center. A retrospective cohort study

**DOI:** 10.1016/j.amsu.2020.01.003

**Published:** 2020-01-15

**Authors:** Emad Hamdy Gad, Yasmin Kamel, Ayman Alsebaey, Anwar Mohammed, Mohammed Alsayed Abdelsamee

**Affiliations:** a Hepatobiliary Surgery, National Liver Institute, Menoufia University, Shebein Elkoum, Egypt; b Hepatology, National Liver Institute, Menoufia University, Shebein Elkoum, Egypt; c Anaesthesia, National Liver Institute, Menoufia University, Shebein Elkoum, Egypt; d Intervention Radiology, National Liver Institute, Menoufia University, Shebein Elkoum, Egypt

**Keywords:** Laparoscopic cholecystectomy, Liver cirrhosis, Harmonic device

## Abstract

With improved laparoscopic techniques, experience, and availability of newer tools and instruments like ultrasonic shears; laparoscopic cholecystectomy (LC) became a feasible option in cirrhotic patients, the aim of this study was to analyze the outcome of LC in cirrhotic patients. Methods: We retrospectively analyzed 213 cirrhotic patients underwent LC, in the period from 2011 to 2019; the overall male/female ratio was 114/99. Results: The most frequent Child-Turcotte-Pugh (CTP) score was A, The most frequent cause of cirrhosis was hepatitis C virus (HCV), while biliary colic was the most frequent presentation. The harmonic device was used in 39.9% of patients, with a significant correlation between it and lower operative bleeding, lower blood and plasma transfusion rates, higher operative adhesions rates, lower conversion to open surgery and 30-day complication rates, shorter operative time and post-operative hospital stays where operative adhesions and times were independently correlated. The 30-day morbidity and mortality were 22.1% and 2.3% respectively while overall survival was 91.5%, higher CTP, and model for end-stage liver disease (MELD) scores, higher mean international normalization ratio (INR) value, lower mean platelet count, higher operative bleeding, higher blood, and plasma transfusion rates, longer mean operative time and postoperative hospital stays were significantly correlated with all conversion to open surgery, 30-day morbidities and mortalities. Conclusion: LC can be safely performed in cirrhotic patients. However, higher CTP and MELD scores, operative bleeding, more blood and plasma transfusion units, longer operative time, lower platelet count, and higher INR values are predictors of poor outcome that can be improved by proper patient selection and meticulous peri-operative care and by using Harmonic scalpel shears.

## Highlights

• LC can be safely performed in cirrhotic patients.
• Outcome is affected by higher CTP and MELD scores.
• Lower platelets count, and higher INR values affect outcome.
• Proper patient selection and peri-operative care improve outcome.
• Harmonic scalpel shears have positive effect on outcome.

## List of abbreviations

AC: Acute cholecystitis
ALB: Albumin
ALT: Alanine transaminase
AST: Aspartate transaminase
BCS: Budd Chiari syndrome
CT: Computerized tomography
CTP: Child-Turcotte-Pugh
FFP: Fresh frozen plasma
GGT: Gama glutamate transferase
GIT: Gastrointestinal tract
HA: Hepatic arterial
HBV: Hepatitis B virus
HCV: Hepatitis C virus
HPB: Hepatopancreatobiliary
INR: International normalization ratio
IOC: Intra-operative cholangiogram
IRB: Institutional review board
LC: Laparoscopic cholecystectomy
LFT: Liver function test
LSC: laparoscopic subtotal cholecystectomy
LT: Liver transplantation
MELD: Model for end-stage liver disease
MD: Mono-polar diathermy
MOF: Multi-system organ failure
NLI: National Liver Institute
PC: Percutaneous transhepatic cholecystostomy
PRBCs: Packed red blood cells
PDS: Polydioxanone
PHG: Portal hypertensive gastropathy
PHN: Portal hypertension
POD: Post-operative day
UE: Upper endoscopy
US: Ultrasonography
UTI: Urinary tract infections

## 1 Introduction

Cholelithiasis in cirrhotic patients has a higher prevalence (2–3 folds) in comparison to the general population due to several reasons (I.e. intravascular hemolysis from hypersplenism, reduced gallbladder motility and emptying due to high estrogen levels, and metabolic liver failure) [[1], [2], [3], [4], [5], [6], [7]].

Despite cirrhosis was previously considered as absolute or relative contraindication for laparoscopic cholecystectomy (LC) due to deaths from postoperative liver failure, sepsis, and hemorrhage [5]; LC became a safe and effective procedure in patients with symptomatic cholelithiasis and liver cirrhosis especially Child-Turcotte-Pugh (CTP) A and B after improved laparoscopic surgery, availability of newer instruments (i.e. ultrasonic shears) and better peri-operative care [8,9], however, it remains a challenging procedure that should be performed by surgeons with experience in both the procedure and the peri-operative management of those patients [3]. There are increased rates of conversion to open surgery, morbidities, and mortalities after LC in cirrhotic patients in comparison to the general population [5,6,10,11]. Those outcomes are affected by several risk factors (i.e. Intra-operative bleeding, transfusion requirements, CTP and Model for end-stage liver disease (MELD) scores) [3]. So, proper patient selection and adequate preoperative optimization of them (i.e. control of ascites, nutritional support, correction of coagulopathy, and upgrading of liver condition) and intra-operative good hemostasis (i.e. materials like oxidized cellulose (Gelfoam), surgicel, and devices like Argon and Harmonic Scalpel) are required for better outcome [6,8].

Furthermore, in cirrhotic patients, the liver becomes fibrotic and stiff with excessive collaterals from portal hypertension (PHN), moreover, the gallbladder tissue becomes woody and friable as patients are presented late in the course of their disease, so, intra-operative meticulous and careful gallbladder dissection by devices like Harmonic Scalpel is required to avoid bleeding and improve outcome in those coagulopathic patients [8]. Moreover, the Harmonic scalpel is an advanced ultrasonic cutting and coagulating surgical device having many advantages (I.e. facilitated dissection, minimal lateral thermal tissue damage, greater precision, and less smoke production) leading to reduced operating time and decreased conversion to open surgery [12,13].

To the best of our knowledge, there is little literature discussing LC in cirrhotic patients regarding predictors of outcome and using Ultrasonic shears, so, our study aimed to analyze this important issue.

## 2 Patients and methods

Two hundred and twenty-five cirrhotic patients underwent LC, in the period from the beginning of 2011 to the beginning of 2019 in the department of hepato-pancreato-biliary (HPB) surgery (tertiary care center), National Liver Institute (NLI), University of Menoufia, Menoufia, Egypt, our study included 213 patients after exclusion of cases who did not complete the follow-up, with data loss, and who refused researches. We did this cohort study which is a single-institution retrospective analysis of a prospectively collected database that assessed these surgical procedures in the period from the beginning of 2011 to mid 2019, where patients were observed from POD1 until the end of June 2019 with a median follow up period of 52 ms, and range of (0.3–98 ms) [14]. The study was approved by our Institutional review board (IRB).

**Fig. 1:**
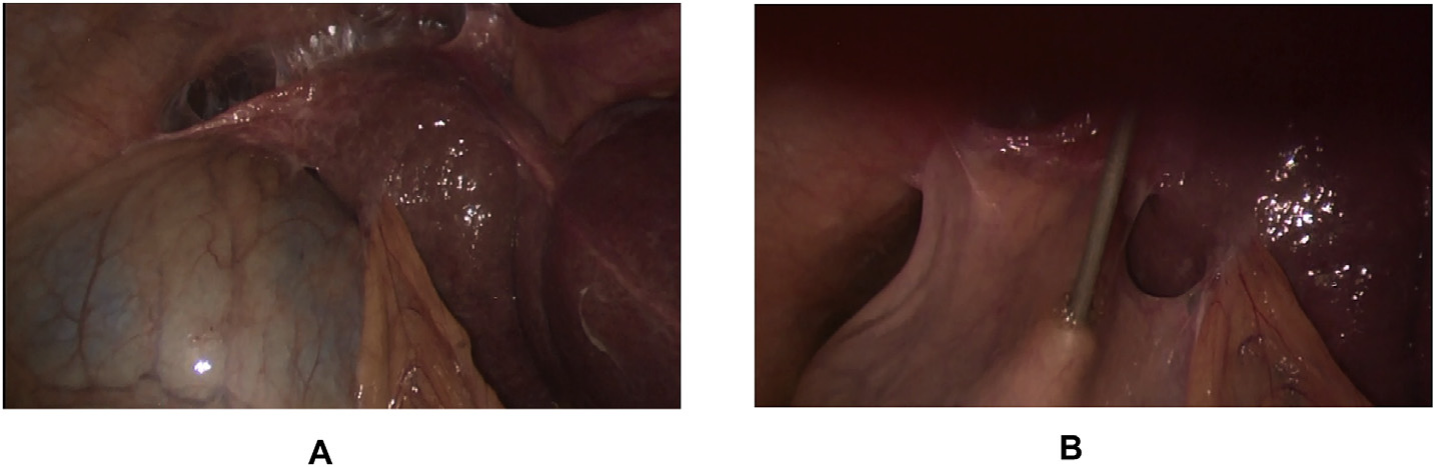
(A): Empyema of gallbladder in early cirrhotic liver, (B): Gallbladder decompression by suction device.

The data were collected from our records in our HPB surgery department where written informed consents were obtained from patients regarding surgeries, and researches. [14] Our work has been reported in line with the STROCSS criteria [15], with researchregistry4590 https://www.researchregistry (see Fig. 1).

**Fig. 2:**
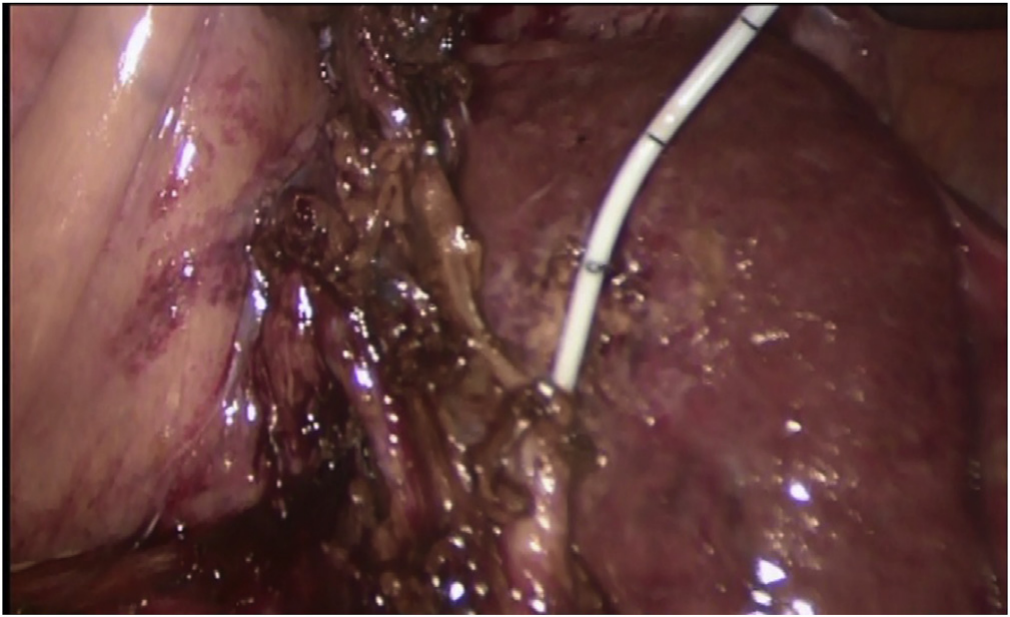
Laparoscopic intraoperativecholangiography

The recorded data included patient demographics, co-morbidities, the diagnosis of liver cirrhosis that was established pre-operatively (i.e. clinically, laboratory data, abdominal ultrasonography (US), and computerized tomography (CT) in some cases) or during operation (i.e. liver gross appearance, and/or liver histology), etiology of liver cirrhosis (i.e. hepatitis C virus (HCV), hepatitis B virus (HBV), …), the diagnosis of cholelithiasis that was established clinically, and confirmed by abdominal US, patient presentation (i.e. biliary colic, acute cholecystitis, …), history or presence of encephalopathy and/or ascites, liver function tests (LFT), coagulation profile, creatinine level, platelet count, CTP and MELD scores, performing pre-operative upper endoscopy (UE) to detect PHN, performing percutaneous transhepatic cholecystostomy (PC), laparoscopic subtotal cholecystectomty (LSC), and intra-operative cholangiography (IOC) Fig. 2, operative adhesions, using Argon and Harmonic devices, operative bleeding, blood, plasma, and platelet transfusion, conversion to open surgery and its causes, operative time per minutes and postoperative hospital stay per days.

### 2.1 Pre-operative patient preparation and surgical techniques

Preoperatively, patients with coagulopathy were given vitamin K, and fresh frozen plasma (FFP) if international normalization ratio (INR) was elevated (given pre-induction), and platelets if platelet count was less than 50,000/μL. Furthermore, control of ascites, nutritional support, and upgrading of the liver condition were done in patients with higher CTP grades. In CTP class late B, and C patients; a conservative treatment was our 1st choice, however, its failure pushed us to do emergency LC with PC trial in some cases. Anesthesia that was performed by the anesthetic author of the manuscript was induced with Diprivan, Fentanyl, muscle relaxant and maintained with inhalation non-hepatotoxic anesthetics (i.e. Isoflurane or Sevoflurane) supplemented with muscle relaxant and Fentanyl. LC and PC were done by the surgical and the intervention radiology authors of the manuscript respectively.

**Fig. 3:**
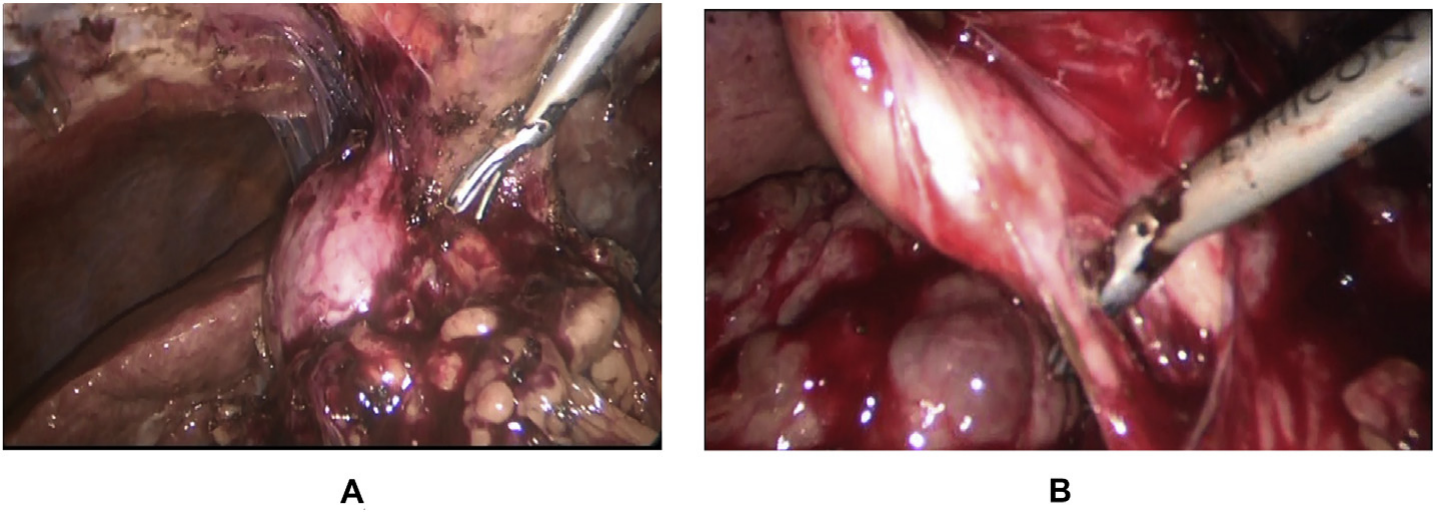
By using Harmonic device: (A): Dissection of omental adhesions to gallbladder, (B): Dissection at Calot's triangle.

**Fig. 4:**
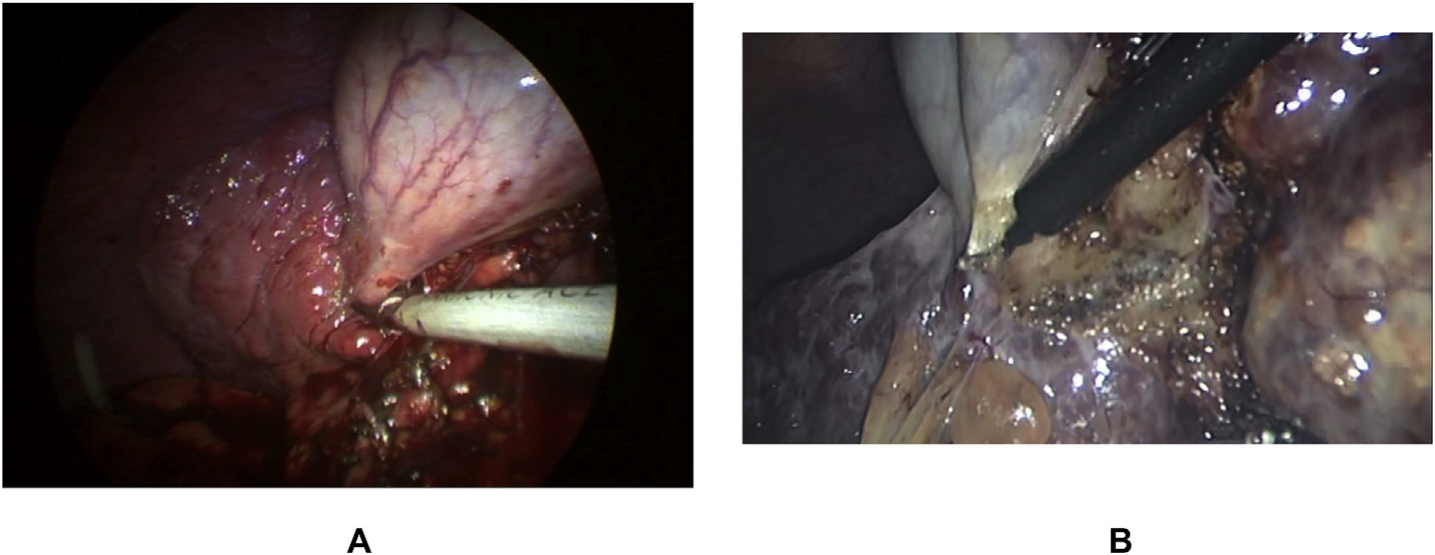
Dissection of the gallbladder from its cirrhotic liver bed by: (A): Harmonic device (B): hook instrument.

**Fig. 5:**
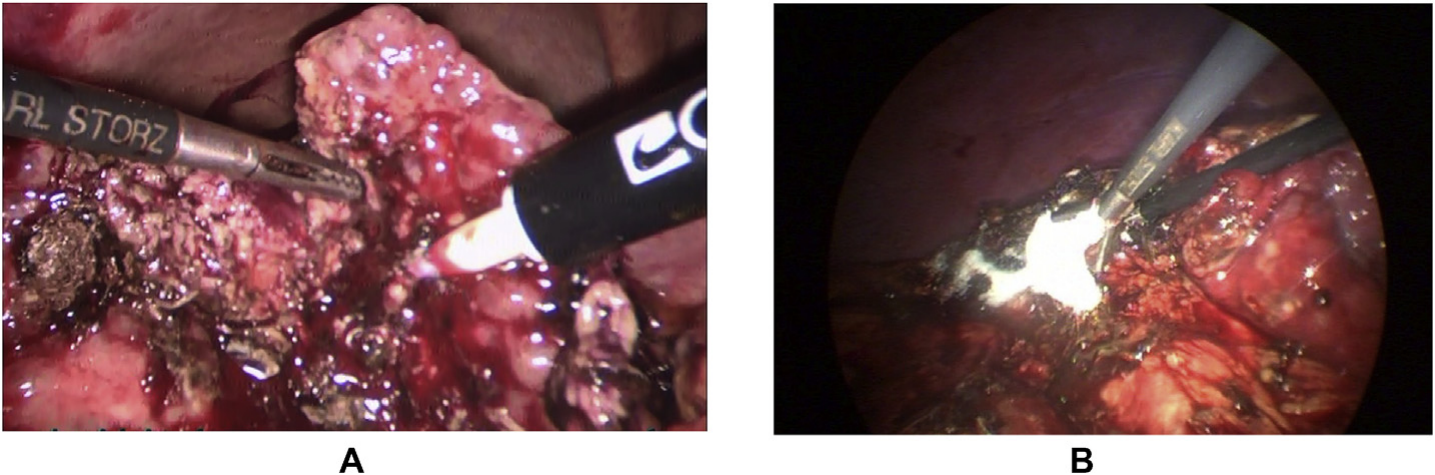
Haemostasis of cirrhotic liver bed by: (A): Argon, (B): Surgicel.

The standard 4-trocar technique was applied for LC maintaining pneumoperitoneum at a pressure of 14 mmHg with some modifications [1]: Application of a 5th port in some cases for elevation of the hypertrophied left lateral liver segment [2], The subxiphoid port was placed more to the right of the midline, while the umbilical port was put on the right, left of the midline or below the umbilicus by open Hasson technique to avoid injury to the falciform ligament recanalized umbilical vein [3].Abdominal wall collaterals were assessed pre-operatively by abdominal CT; moreover, *trans*-illumination of the abdominal wall during port placement was done to identify these collaterals preventing catastrophic bleeding [4], Avoiding excessive traction to prevent avulsion of gallbladder from its bed, aspiration of gallbladder content (empyema or mucocele) to facilitate its grasping, and avoiding blunt dissection Fig. 1 [5] careful dissection by using either Harmonic device (Ethicon Endo-Surgery, Cincinnati, OH) in some cases (its use was according to its availability or intra-operative findings (i.e. numerous collaterals and/or marked adhesions)), Fig. 3, Fig. 4A or monopolar electrocautery in other cases, Fig. 4B moreover, good hemostasis of gallbladder bed was done by using Argon device, Fig. 5Aoxidized cellulose (Gelfoam) (Pfizer, New York, NY), surgicel (Johnson & Johnson, New Brunswick, NJ) Fig. 5B and/or radiofrequency ablation (Habeeb sealer), furthermore, preparation of sufficient amount of FFP, platelets, and packed red blood cells (PRBCs) was done to be used when needed.

When Harmonic device was used and after its division of omental adhesions to the gallbladder and the liver surface, dissection at Calot's triangle was done by its setting at level 2 (i.e. minimum, for less cutting and more coagulation), then after Harmonic closure of the cystic artery and duct and their division, enforcement was done with metallic clips or Endoloop PDS (Ethicon), then after its setting at level 5 (i.e. maximum, for more cutting and less coagulation), dissection of the GB from its bed was done. Fig. 3, Fig. 4A [16].

LSC was performed in some cases due to marked gallbladder adhesions and/or difficult dissection of it from its bed to avoid vigorous bleeding and biliary injuries; furthermore, it was classified according to Palanivelu et al., 2006 [2] as: (LSC I: leaving the gallbladder posterior wall intact with the liver with cauterization of the remnant mucosa, LSC II: circumferential division of the infundibulum as close to the junction of the GB and the cystic duct as safely as possible followed by cauterization of the mucosa in the proximal remnant and closure of the flap with continuous PDS 3/0 (Ethicon) suturing or with Endoloop PDS (Ethicon), LSC III: a combination of LSC I and LSC II) [2].

Patient were followed-up daily during hospital stay until discharge, then weekly in the out-patient clinic until the end of the 1st post operative month by clinical assessment, laboratory (i.e. LFT, ….), US, and others if needed (i.e. CT) to detect 30-days morbidities, and mortalities, furthermore, they were followed-up in the out-patient clinic until the end of the follow-up period to detect long-term cirrhosis related mortalities, and overall survival, moreover, comparison between patients was done regarding Harmonic device use, conversion to open surgery, 30-days morbidities, and mortalities.

Statistical analysis: All data were tabulated and processed with SPSS software (Statistical Product and Service Solutions, version 21, SSPS Inc, Chicago, IL, USA) and Windows XP (Microsoft Corporation, Redmond, Washington, USA). Qualitative data were expressed in frequency and percentage and analyzed with the chi-square or Fisher exact tests. Quantitative data were expressed as the mean and standard deviation or median and range and were compared with the Student t or Mann- Whitney U tests. Comparison between patients regarding Harmonic device use, conversion to open surgery, 30-day morbidities and mortalities were done using Univariate and then multivariate analyses. The Kaplan–Meier method was applied for survival analysis. In all tests, a P value of <0.05 was considered significant.

## 3 Results

### 3.1 Characteristics of patients

They were classified as 114 (53.5%) males, and 99 (46.5%) females; their mean age was 44.3 ± 9.5 years. Co-morbidities affected 22.1% of patients while HCV infection was the most frequent cause of liver cirrhosis (85%). Acute cholecystitis (AC), biliary colic, and gall stone pancreatitis were the presentations in 26.3%, 68.1%, and 5.6% of them respectively. Patients were classified as CTP score A, B, and C in 59.6%, 36.2%, and 4.2% of them respectively while their mean MELD score and CTP NO were 9.8 ± 3.7 and 6.4 ± 1.5 respectively. Pre-operative UE was performed in 65.3% of patients that showed varices and portal hypertensive gastropathy (PHG) in 23% and 6.6% of them respectively. PC and IOC were performed in 4 (1.9%) and 19 (8.9%) of them respectively. Intra-operative adhesions were noticed in 21.1% of them. Argon and harmonic devices were used in 34 (16%), and 85 (39.9%) of them respectively. LSC was done in 9 (4.2%) of patients. The operative bleeding affected 39% of patients where blood and plasma transfusions were given to 9.4% and 34.7% of them respectively. Eight (3.8%) of patients were converted to open surgery due to unclear anatomy from inflammations and adhesions (1 patient), bleeding (2 patients), biliary, and gastric injuries (5 patients). Lastly, the mean operative time and postoperative hospital stays were 109.3 ± 55.4 min, and 2.4 ± 3 days respectively Table 1.

**Table 1:** Characteristics of patients.

Character	(Mean ± SD) or No213	(%) (100%)
Gender		
Males	114	(53.5%)
Females	99	(46.5%)
Age (years) (Mean ± SD)	44.3 ± 9.5	
Co-morbidity	47	(22.1%)
Cirrhosis cause		
HCV	181	(85%)
HBV	18	(8.5%)
Cryptogenic	10	(4.7%)
BCS	4	(1.9%)
Presentation		
AC	56	(26.3%)
Biliary colic	145	(68.1%)
Gall stone Pancreatitis	12	(5.6%)
Ascites		
No	205	(96.2%)
Mild	6	(2.8%)
Moderate	2	(0.9%)
Encephalopathy	0	
AST (U/L) (Mean ± SD)	33.5 ± 13.3	
ALT (U/L) (Mean ± SD)	36.2 ± 13.8	
Alkaline phosphatase (U/L) (Mean ± SD)	84.8 ± 45.7	
GGT (U/L) (Mean ± SD)	42.2 ± 14.5	
Alb (g/dL) Mean ± SD)	3.2 ± 0.7	
INR (Mean ± SD)	1.3 ± 0.3	
Creatinine (mg/dL) (Mean ± SD)	0.7 ± 0.2	
Platelet count (1000/μL) (Mean ± SD), (Median (range))	254.9 ± 99.2	
	260 (50–450)	
CTP score		
A	127	(59.6%)
B	77	(36.2%)
Early B*	61	(28.7%)
Late B*	16	(7.5%)
C	9	(4.2%)
CTP NO(Mean ± SD)	6.4 ± 1.5	
MELD score (Mean ± SD)	9.8 ± 3.7	
Pre-operative upper endoscopy	139	(65.3%)
PC Trial	4	(1.9%)
IOC	19	(8.9%)
Operative adhesions	45	(21.1%)
Using argon device	34	(16%)
Using Harmonic device	85	(39.9%)
LSC	9	(4.2%)
Operative bleeding	83	(39%)
Blood transfusion	20	(9.4%)
Plasma transfusion	74	(34.7%)
Platelets transfusion	0	(0%)
Conversion to open	8	(3.8%)
Operative time (min) (Mean ± SD)	109.3 ± 55.4	
Hospital stay (postoperative) (days) (Mean ± SD)	2.4 ± 3	

HCV: Hepatitis C virus, HBV: Hepatitis B virus, BCS: Budd Chiari syndrome, AC: Acute cholecystitis, AST: Aspartate transaminase, ALT: Alanine transaminase, GGT: Gama glutamate transferase, Alb: Albumin, INR: International normalization ratio, CTP: Child-Turcotte-Pugh, Early B*: scores 7,8, Late B*: score 9, MELD: Model for end-stage liver disease, PC: percutaneous cholecystostomy, IOC: Intraoperative cholangiogram, LSC: laparoscopic subtotal cholecystectomy.

### 3.2 The outcome of patients

**Fig. 6:**
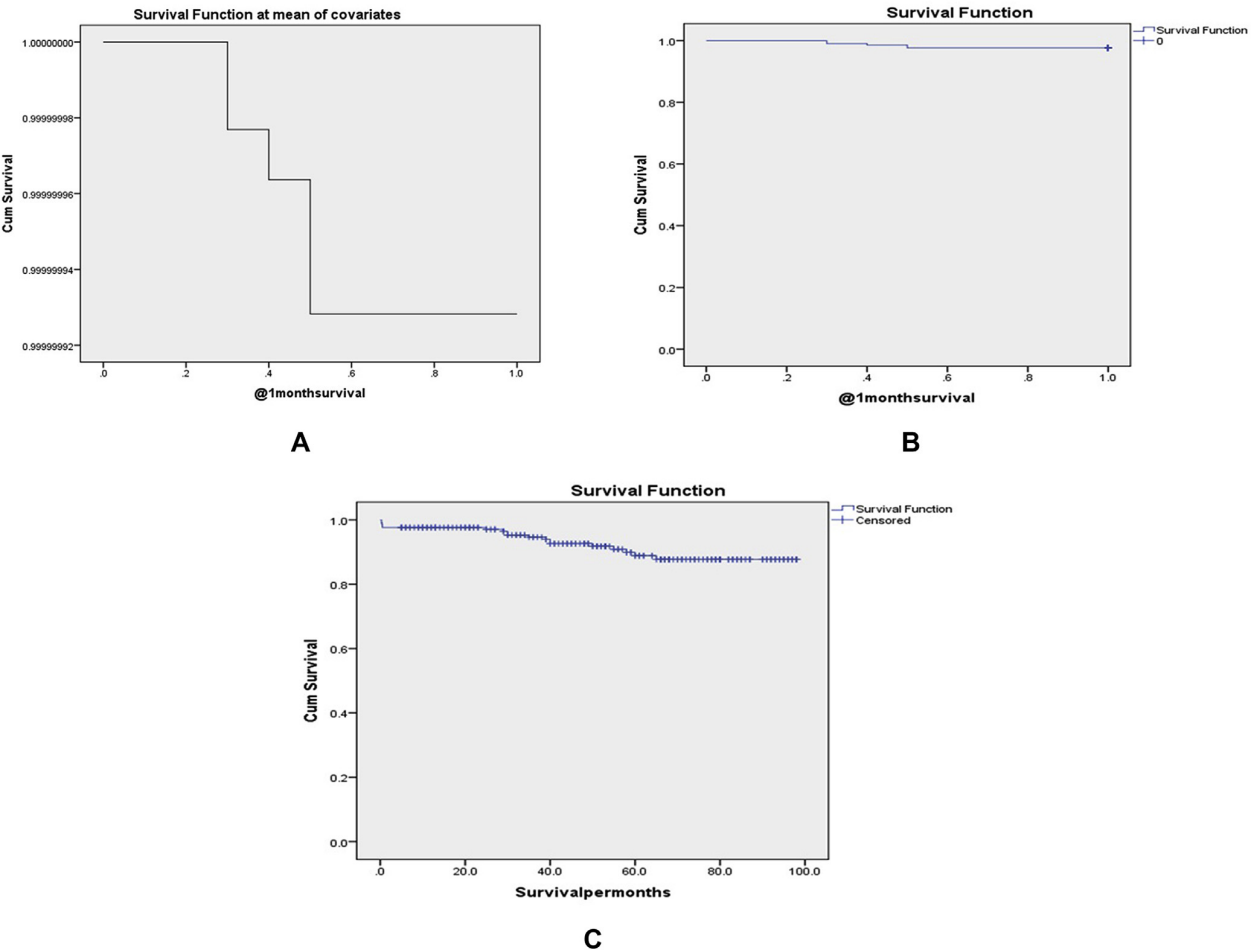
(A) Cox Regression 1-month survival curve (B) Kaplan-Meier 1-month survival curve (C) Kaplan-Meier overall survival curve.

Forty-seven (22.1%) of patients were complicated with one or more intra-or post-operative complication in the 1st post-operative month. Biliary and gastric injuries affected 2 (0.9%), and 3 (1.4%) of patients respectively (i.e. biliary injuries were Strasberg D, while gastric injuries were full thickness ones in the anterior gastric body), those patients were converted to open surgery to do a primary repair of these injuries with a successful outcome (Clavien grade III). The postoperative infection affected 30 (14.1%) of patients in the form of chest (6.6%), wound (4.2%), and urinary tract infections (UTI) (5.2%), All those infections improved after conservative management with antibiotics therapy except 2 cases with chest infection that were complicated with pneumonia, and sepsis and died (Clavien II, V). Thirteen (6.1%) of patients were complicated with postoperative liver decompensation as follow: gastrointestinal tract (GIT) bleeding affected 2 patients where one of them improved after endoscopic band ligation (Clavien III) and the other one died from massive bleeding and multi-system organ failure (MOF) (Clavien V), however, ascites affected 11 patients where 9 of them improved with medications (Clavien II) but the other 2 patients died from liver failure (Clavien V), on the other hand, hepatic encephalopathy complicated 4 patients where 2 of them improved with anti-coma measures (Clavien II), but the other 2 patients died from liver failure (Clavien V), lastly, post-operative cholestasis affected 4 patients where 2 of them improved with liver support (Clavien II), and the other 2 patients died from liver failure (Clavien V). The operative site hematoma and port site bleeding affected 6 (2.8%) and 8 (3.8%) of our patients respectively, where they were managed successfully with conservative treatment (Clavien II). The 30-day mortality affected 5 (2.3%) of our patients due to Pneumonia and sepsis (2 patients), liver failure (2 patients), and GIT bleeding (one patient). The long-term cirrhosis-related mortality was the late insult in 13 (6.1%) of our cases. Lastly, the overall survival during the follow-up period in our series was 91.5%. Table 2, Fig. 6.

**Table 2:** Outcome of patients.

Character	No 213	(%) (100%)	Clavien grade of complications
30-day complications	47	(22.1%)	
Biliary injury	2	(0.9%)	III
Gastric injury	3	(1.4%)	III
Postoperative infection	30	(14.1%)	
Chest infection	14	(6.6%)	II, V
Wound infection	9	(4.2%)	II
UTI	11	(5.2%)	II
Postoperative decompensation	13	(6.1%)	
GIT bleeding	2	(0.9%)	III, V
Ascites	11	(5.2%)	II, V
Encephalopathy	4	(1.8%)	II, V
Cholestasis	4	(1.8%)	II, V
Operative site hematoma	6	(2.8%)	II
Port side bleeding	8	(3.8%)	II
30-day mortality	5	(2.3%)	
Causes:			
Pneumonia, sepsis	2	(0.9%)	
Liver failure	2	(0.9%)	
GIT bleeding	1	(0.5%)	
Long-term mortality (Cirrhosis related)	13	(6.1%)	
Overall survival	195	(91.5%)	
Survival (months)			
Mean ± SD	51.1 ± 27.2		
Median (Range)	52	(0.3–98)	

UTI: Urinary tract infection, GIT: Gastrointestinal.

### 3.3 Comparison between patients regarding harmonic device use

On univariate analysis, there was a significant correlation between Harmonic device and the followings: Higher operative adhesions rates, lower operative bleeding, lower blood and plasma transfusion rates, lower argon use, non-conversion to open surgery, lower 30-day complication rates, shorter mean operative time and postoperative hospital stays. On the other hand, on multivariate analysis, there was an independent correlation between it and both operative adhesions and shorter operative time. Table 3.

**Table 3:** Comparison between patients regarding Harmonic device use.

Character	Harmonic device use (No = 85)	No Harmonic device (No = 128)	P value Univariate analysis	P value Multivariate analysis
Operative bleeding	5 (5.9%)	78 (60.9%)	0.000	>0.05
Blood transfusion	1 (1.2%)	19 (14.8%)	0.000	>0.05
Plasma transfusion	5 (5.9%)	69 (53.9%)	0.000	>0.05
Operative adhesions	27 (31.8%)	18 (14.1%)	0.002	0.000
Putting drain	4 (4.7%)	70 (54.7%)	0.000	>0.05
Argon use	2 (2.4%)	32 (25%)	0.000	>0.05
Operative time (min) (Mean ± SD)	69.2 ± 22.8	135.9 ± 54.8	0.000	0.000
Conversion to open	0	8 (6.3%)	0.016	>0.05
Hospital stay (days) (Mean ± SD)	1.3 ± 0.9	3.1 ± 3.6	0.000	>0.05
30-day complications	4 (4.7%)	43 (33.6%)	0.000	0.052
30-day mortality	0	5 (3.9%)	0.3	>0.05

On reviewing the literature of LC in cirrhotic patients, the conversion to open surgery, morbidity and mortality rates ranged from 0 to 17%, 0–52%, and 0–8% respectively, however, the operative time and postoperative hospital stay were in the range of 50.4–155 min, and 1–7.3 days respectively. Table 4. It is clear from the table that our values are within the previous ranges.

**Table 4:** Laparoscopic cholecystectomy in cirrhotics: a Literature review.

Author	Year	Patients NO	CTP A	CTP B	CTP C	Conversion No %	Operative time minutes	Morbidity No %	Hospital stay days	Mortality No %
Lacy et al. [17]	1995	11	7	3	1	1 9%	92	0	1.8	0
Gugenheim et al. [18]	1996	9	9	0	0	0	–	2 22%	3	0
Yerdel et al. [19]	1997	7	6	0	1	0	155	0	6.7	0
Sleeman et al. [20]	1998	25	16	9	0	–	116	8 32%	1.7	0
Friel et al. [21]	1999	30	23	7	0	5 17%	139	7 23%	3.1	0
Morino et al. [22]	2000	33	27	4	2	2 6%	114	0	2.8	0
Clark et al. [23]	2001	25	14	9	2	0	107	13 52%	4	1 4%
Yeh et al. [24]	2002	226	193	33	0	10 4.4%	87	15 6.6%	4.7	2 0.8%
Cucinotta et al. [25]	2003	22	12	10	0	2 9%	115	8 36%	5	0
Cobb et al. [26]	2004	50	39	10	1	2 4%	155	8 16%	3	0
Ji et al. [27]	2005	38	19	15	4	2 5%	63	5 13%	4.6	0
Curro et al. [28]	2005	42	22	16	4	3 7%	128	15 35%	7.3	2 5%
Palanivellu et al. [2]	2006	265	–	–	0	2 0.75%	65	109 41%	4	0
Curro et al. [29]	2007	50	32	18	0	2 4%	105	12 24%	5	0
Cappellani et al. [30]	2008	40	20	10	10	3 8%	111	10 25%	6	3 8%
Pavlidis et al. [10]	2009	38	29	9	0	6 15.7%	–	3 7.8%	4.4	0
Delis et al. [5]	2010	220	194	26	0	12 6%	95	42 19%	4	0
Bessa et al. [31]	2011	40	27	13	0	3 7.5%	–	13 32.5%	2	0
Lledo et al. [32]	2011	43	26	15	2	5 7.2%	84.3	16 37.2%	3.1	0
Nguyen et al. [7]	2011	68	47	19	2	4 5.9%	120	5 7.4%	1	0
Quillin III et al. [3]	2012	94	63	20	2	10 11%	124	32 34%	2.6	4 4%
Khan and Siddiq [33]	2013	82	46	24	12	3 3.7%	80	41 50%	3.2	0
Hassan [34]	2014	71	–	–	0	–	50.4	–	1.8	–
Kassem [16]	2018	62	35	27	0	2 3.2%	72.9	1 1.6%	1.4	0
Present study	2019	213	127	77	9	8 3.8%	109.3	47 22.1%	2.4	5 2.3%

-: means unknown.

### 3.4 Predictors of conversion to open surgery

On univariate analysis, conversion to open surgery was associated with the following: CTP score C, and B and higher mean CTP NO, higher mean MELD score, and INR values, lower mean platelet count, higher operative adhesions, higher bleeding, blood and plasma transfusion rates, absent Harmonic device use, higher argon use, and higher 30-day complication rates, longer mean operative times and postoperative hospital stays. However, on multivariate analysis, there was no independent correlation between any variable and conversion. Table 5.

**Table 5:** Predictors of conversion to open surgery.

Character	Conversion No = 8	No conversion No = 205	P value Univariate analysis	P value Multivariate analysis
Age (years) (Mean ± SD)	40.3 ± 5.4	44.4 ± 9.6	0.2	
Gender Males Females	5 (62.5%) 3 (37.5%)	109 (53.2%) 96 (46.8%)	>0.05	
Co-morbidity	2 (25%)	45 (22%)	>0.05	
Presentation AC Biliary colic Gall stone Pancreatitis	5 (62.5%) 3 (37.5%) 0	51 (24.9%) 142 (69.3%) 12 (5.9%)	0.06	>0.05
CTP score A B C	0 7 (87.5%) 1 (12.5%)	127 (62%) 70 (34.1%) 8 (3.9%)	0.002	>0.05
CTP No(Mean ± SD)	8.1 ± 1.5	6.3 ± 1.5	0.01	>0.05
MELD score (Mean ± SD)	14.6 ± 2.3	9.6 ± 3.6	0.000	>0.05
INR	1.7 ± 0.4	1.3 ± 0.3	0.008	>0.05
Platelet count (1000/μL) (Mean ± SD)	150 ± 71.7	259 ± 98	0.003	>0.05
Operative adhesions	8 (100%)	37 (18%)	0.000	>0.05
Operative bleeding	8 (100%)	75 (36.6%)	0.000	>0.05
Blood transfusion	3 (37.5%)	17 (8.3%)	0.03	>0.05
Plasma transfusion	8 (100%)	66 (32.2%)	0.000	>0.05
IOC	0	19 (9.3%)	>0.05	
Harmonic use	0	85 (41.5%)	0.02	>0.05
Argon use	5 (62.5%)	29 (14.1%)	0.003	>0.05
Operative time (min) (Mean ± SD)	208.8 ± 21	105.4 ± 52.7	0.000	>0.05
Hospital stay (days) (Mean ± SD)	10.3 ± 2.9	2.1 ± 2.5	0.000	>0.05
30-day complications	8 (100%)	39 (19%)	0.000	>0.05
30-day mortality	1 (12.5%)	4 (2%)	0.2	

AC: Acute cholecystitis, CTP: Child-Turcotte-Pugh, MELD: Model for end-stage liver disease, INR: International normalization ratio, IOC: Intraoperative cholangiogram.

### 3.5 Predictors of 30-day morbidity

On univariate analysis, the following factors were significantly associated with 30-day complication: AC as presentation, CTP scores C, and B, higher mean CTP NO, INR value, and MELD score, lower mean platelet count, higher operative adhesions, higher bleeding, blood and plasma transfusion rates, lower rate of harmonic use and higher rate of argon use, longer mean operative time and postoperative hospital stay. However, on multivariate analysis, there was no independent association between morbidity and any previous variable. Table 6.

**Table 6:** Predictors of 30-day morbidity.

Character	30-day morbidity No = 47	No morbidity No = 166	P value Univariate analysis	P value Multivariate analysis
Age (years) (Mean ± SD)	43.4 ± 9.2	44.5 ± 9.6	>0.05	
Gender Males Females	23 (48.9%) 24 (51.1%)	91 (54.8%) 75 (45.2%)	>0.05	
Co-morbidity	11 (23.4%)	36 (21.7%)	>0.05	
Presentation AC Biliary colic Gall stone Pancreatitis	35 (74.5%) 10 (21.3%) 2 (4.3%)	21 (12.7%) 135 (81.3%) 10 (6%)	0.000	>0.05
CTP score A B C	2 (4.3%) 36 (76.6%) 9 (19.1%)	125 (75.3%) 41 (24.7%) 0	0.000	>0.05
CTP NO(Mean ± SD)	8.2 ± 1.6	5.9 ± 1	0.000	>0.05
MELD score (Mean ± SD)	14.6 ± 2.8	8.4 ± 2.6	0.000	>0.05
INR	1.7 ± 0.3	1.2 ± 0.2	0.000	>0.05
Platelet count (1000/μL) (Mean ± SD)	148.9 ± 79.3	284.9 ± 82.4	0.000	>0.05
Operative adhesions	20 (42.6%)	25 (15.1%)	0.000	>0.05
Operative bleeding	44 (93.6%)	39 (23.5%)	0.000	>0.05
Blood transfusion	15 (31.9%)	5 (3%)	0.000	>0.05
Plasma transfusion	39 (83%)	35 (21.1%)	0.000	>0.05
Harmonic use	4 (8.5%)	81 (48.8%)	0.000	>0.05
Argon use	23 (48.9%)	11 (6.6%)	0.000	>0.05
Operative time (min) (Mean ± SD)	172.1 ± 47.1	91.5 ± 43.4	0.000	>0.05
Hospital stay (days) (Mean ± SD)	6.9 ± 3.8	1.2 ± 0.4	0.000	>0.05

AC: Acute cholecystitis, CTP: Child-Turcotte-Pugh, MELD: Model for end-stage liver disease, INR: International normalization ratio.

### 3.6 Predictors of 30-day mortality

On univariate analysis, 30-day mortality was associated with the following: AC as presentation, CTP score C, higher mean CTP NO, INR value, and MELD score, lower mean platelet count, higher operative bleeding, blood, plasma transfusion, and 30-day complication rates, longer mean operative time and postoperative hospital stay. However, on multivariate analysis, there was no independent correlation between any variable and mortality. Table 7, Fig. 6.

**Table 7:** Predictors of 30-day mortality.

Character	30-day mortality No = 5	No mortality No = 208	P value Univariate analysis	P value Multivariate analysis
Age (years) (Mean ± SD)	49.2 ± 13.5	44.1 ± 9.4	>0.05	
Gender Males Females	4 (80%) 1 (20%)	110 (52.9%) 98 (47.1%)	>0.05	
Co-morbidity	0	47 (22.6%)	>0.05	
Presentation AC Biliary colic Gall stone Pancreatitis	5 (100%) 0 0	51 (24.5%) 145 (69.7%) 12 (5.8%)	0.001	>0.05
CTP score A B C	0 1 (20%) 4 (80%)	127 (61.1%) 76 (36.5%) 5 (2.4%)	0.000	>0.05
CTP NO	10.6 ± 0.9	6.3 ± 1.4	0.000	0.3
MELD score (Mean ± SD)	18 ± 1.7	9.6 ± 3.5	0.000	0.3
INR	2.1 ± 0.3	1.3 ± 0.3	0.004	0.4
Platelet count (1000/μL) (Mean ± SD)	75 ± 14.1	259.2 ± 96.3	0.000	>0.05
Operative bleeding	5 (100%)	78 (37.5%)	0.008	>0.05
Blood transfusion	4 (80%)	16 (7.7%)	0.000	0.5
Plasma transfusion	5 (100%)	69 (33.2%)	0.005	>0.05
Harmonic use	0	85 (40.9%)	0.08	>0.05
Argon use	2 (40%)	32 (15.4%)	0.2	
Conversion to open	1 (20%)	7 (3.4%)	0.2	
Operative time (min) (Mean ± SD)	224 ± 20.7	106.5 ± 53	0.000	0.3
30-day complications	5 (100%)	42 (20.2%)	0.000	>0.05
Hospital stay (days) (Mean ± SD)	11.6 ± 2.6	2.2 ± 2.6	0.001	>0.05

AC: Acute cholecystitis, CTP: Child-Turcotte-Pugh, MELD: Model for end-stage liver disease, INR: International normalization ratio.

## 4 Discussion

LC in the cirrhotic patient is considered a challenging procedure that should be done by surgeons experienced in both the operation and the peri-operative dealing with liver cirrhosis. Before LC, patients should be informed about the risks of surgery in cirrhotic patients^.^ [3] In comparison to the general population, LC in cirrhotics is associated with more bleeding and worse outcome. Hemorrhage may result from abdominal wall varices from PHN, coagulopathy due to the low synthesis of coagulation factors and thrombocytopenia secondary to hypersplenism [5,28]^.^ To avoid and/or decrease bleeding and improve such outcome, we followed certain pre-, and intraoperative measures: Preoperatively, we performed proper selection, and optimization of our patients by choosing patients with CTP class A, and early B, correction of coagulopathy by FFP and vitamin K injection when INR was elevated (we did not give platelets as our patients platelets were more than 50,000/μL). In patients with higher CTP scores (i.e. Late B, and C), control of ascites, good nutritional support, reduction of portal venous pressure, and liver support were done to upgrade their liver condition for better postoperative outcome, however, patients with persistent higher scores (16 patients CTP class late B, and 9 patients class C), were presented with AC where aggressive conservative treatment was tried for them to avoid suspected LC related massive bleeding, liver failure, and poor outcome, and to decrease bleeding from adhesions during their future liver transplantation (LT) procedures as they were candidates for LT in our LT program, but unfortunately, the conservative management failed, and LC was performed for them. In similar Curro et al., 2005 [28] and Lledo’ et al., 2011 [32] performed LC in their CTP class C patients presented with AC after the failure of conservative treatment.

As a less invasive procedure, PC was tried in 4 of our patients with CTP class C presented with AC after the failure of conservative treatment but unfortunately failed and we were pushed to do LC for them. On the other hand Curro et al., 2005 [28] and Pessaux et al., 2000 [35] performed PC successfully in their CTP class C patients. Similarly, Quillin III et al., 2013 [3]^,^ Pavlidis et al., 2009 [10]^,^ Lledo’ et al., 2011 [32]^,^ and Agresta1 et al., 2015 [36] suggested PC for CTP class C patients.

Intra-operatively, we followed certain protocols to avoid and/or decrease bleeding as mentioned previously (i.e. Putting the subxiphoid port more to the right of the midline, putting the umbilical port on the right, left of the midline or below the umbilicus by open Hasson technique to avoid injury to the falciform ligament recanalized umbilical vein, … …).

Argon beam coagulation was used in 34 (16%) of our patients to control excessive oozing from the gallbladder bed during its removal, also, Nguyen et al., 2011 [7] and Bessa et al., 2011 [31] used it for perfect hemostasis; however, Argon was significantly associated with non Harmonic use, higher conversion and morbidity rates in our study. The explanation for this is that Argon was significantly related to operative bleeding (P = 0.000), that was significantly associated with those parameters.

Harmonic device is an ultrasonic surgical instrument having five effects: grasping, coagulation, cavitations, cutting, and dissection with some advantages during LC (i.e. minimal lateral thermal tissue damage, reduced ligature demand, increased patient safety, no smoke, no charring, no debris accumulation, greater precision near vital structures, better visibility in the surgical field, and more hemostatic and biliostatic support) [13,37].

In cirrhotics, Harmonic device facilitates bloodless detachment of the gallbladder from its bed and saves dissection of the structures at the hilum where there are multiple portal hypertensive collaterals, neovascularizations and vascularized adhesions (i.e. omental adhesions to the gallbladder or the liver surface ….) [6,28]. Similarly, Harmonic instrument that was used in 39.9% of our patients mainly due to marked adhesions (independent correlation) was independently associated with shorter operative time. The explanation for this is its significant association with lower rates of operative bleeding and argon use, as well as its significant relation to non conversion to open surgery and lower rates of putting drains that lead to shorter surgical duration. Also, Power et al., 2000 [12] demonstrated shorter surgical time and minimal blood loss, as well as a low conversion when Harmonic was used in their LC cases. Moreover, using harmonic during LC for AC had a significantly less blood loss and lower conversion to open surgery in comparison to mono-polar diathermy (MD) in Catena et al., 2014 [13] study. On the other hand, using clip-less Harmonic during LC had good impact on operative time, blood loss and conversion rate in Kassem and Hassouna, 2018 [16] study, and on operative time in Bessa et al., 2008 [38] study.

LSCs in cirrhotics are used to avoid visceral, biliary, and vascular injuries and to avoid massive bleeding from dissection at the risky porta hepatis and at the cirrhotic liver bed [39]. It is a feasible and safe procedure utilized in difficult complicated cases to avoid conversion to open surgery with acceptable results [[40], [41], [42]]. It was performed in 9 (4.2%) of our patients (4 CTP class late B, and 5 CTP class C) and succeeded in avoiding intra-operative mortality from massive bleeding and in avoiding biliary injuries despite 2 post-operative mortalities from liver failure, furthermore, it was classified according to Palanivelu et al., 2006 [2]^,^ into 7 cases LSC I, and 2 cases LSC II, Similarly, It was recommended by Machado, 2012 [6] to avoid uncontrollable bleeding from gallbladder bed (LSCI), to avoid risky hilum (LSCII) or both (LSCIII), furthermore, it was successfully performed in 7.9%,11.5%, 14% and 19.4% of patients in Ji et al., 2004 [27]^,^ Tuech et al., 2002 [43]^,^ Lledo’ et al., 2011 [32]^,^ and Kassem and Hassouna, 2018 [16] studies respectively.

Despite increased experience and improvements in laparoscopic surgery and technology, conversion from LC to open surgery remains a matter of concern; however, It is not a complication, but a way for preventing more dangerous catastrophes, it can be divided into elective conversions (unclear anatomy from adhesions or inflammations, difficult dissection, advanced pathology), or enforced (emergent) conversions (serious operative complication; bleeding, vascular, visceral or biliary injuries) [9,13,44,45]. Our LC in cirrhotics conversion rate (3.8%) was within the previous literature range of conversions (0–17%), it was due to unclear anatomy, bleeding, and visceral injuries, similarly, anatomic distortion, uncontrollable bleeding, and biliary injury were conversion causes in Huscher et al., 2003 [37] study, however, unclear anatomy and uncontrollable bleeding were its reasons in Quillin III et al., 2013 [3]^,^ Delis et al., 2010 [5], El-Awadi et al., 2009 [9], Pavlidis et al., 2009 [10], Power et al., 2000 [12], Catena et al., 2014 [13], Cappellani et al., 2008 [30], Lledo’ et al., 2011 [32], and Khan and Siddiq, 2013 [33] studies, however, uncontrollable bleeding was its cause in Kassem and Hassouna, 2018 [16] and Curro et al., 2005 [28] studies. On the other hand, surgeon inexperience and immobile liver were the reasons for it in Schiff et al., 2005 [8] study.

There are several known predictors of conversion in general population (i.e. liver cirrhosis, morbid obesity, old age, male gender, previous upper abdominal surgery, gallbladder wall thickness, and emergency LC) [44,46], however, higher MELD, and CTP scores, as well as bleeding, were our predictors of conversion in cirrhotic patients. Similarly, higher MELD score was predictor of it in Quillin III et al., 2013 [3], and Delis et al., 2010 [5] studies, while higher CTP score was its predictor in Nguyen et al., 2011 [7] study, however, blood loss was its independent predictor in Quillin III et al., 2013 [3] study. On the other hand, CTP score was not associated with it in Quillin III et al., 2013 [3], Delis et al., 2010 [5], and Khan and Siddiq, 2013 [33] studies.

The increased risk of post-operative infection in cirrhotic patients is explained by diminished Kupffer cell action from impaired liver function leading to endotoxinemia, infection and sepsis [10,23]. Similarly, post-operative infection was our most frequent complication (14.1%), also, it was the most frequent morbidity in Quillin III et al., 2013 [3], Nguyen et al., 2011 [7], El-Awadi et al., 2009 [9], Power et al., 2000 [12], Clark et al., 2001 [23], Curro et al., 2005 [28], and Cappellani et al., 2008 [30] studies.

The cirrhotic liver has poor compensation to the decreased hepatic arterial (HA) blood flow and liver ischemia from the anesthesia and to the released inflammatory mediators from this ischemia, leading to liver failure and MOF [10]. In similar, in our work, postoperative liver decompensation was the 2nd most frequent morbidity (6.1%), also, it was the 2nd most frequent complication in Palanivelu et al., 2006 [2], Quillin III et al., 2013 [3], El-Awadi et al., 2009 [9], Sleeman et al., 1998 [20], Cappellani et al., 2008 [30], and Lledo’ et al., 2011 [32] studies.

Higher CTP and MELD scores, higher INR values, more bleeding, higher blood and plasma transfusion rates, and lower mean platelet count were associated with morbidity in our series. Similarly, higher CTP, and MELD scores, increased INR and NO of transfused PRBCs were associated with morbidity in Quillin III et al., 2013 [3] study, while higher CTP score was associated with morbidity in Curro et al., 2005 [28] and Angrisani et al., 1997 [47] studies, however, higher MELD score was associated with complications in Delis et al., 2010 [5] study, while increased INR, and decreased platelet count were associated with morbidity in Perkins et al., 2004 [48] Study. In contrast, CTP score was not associated with morbidity in Delis et al., 2010 [5], Nguyen et al., 2011 [7] and Clark et al., 2001 [23] studies.

Liver failure, sepsis, and GIT bleeding were the causes of our patient mortalities, similarly, liver failure and sepsis were causes of death in Machado, 2012 [6] and Curro et al., 2005 [28] studies, on the other hand, liver failure and variceal bleeding were causes of it in Cappellani et al., 2008 [30] study, however, liver failure was a cause of it in Quillin III et al., 2013 [3] study, lastly, sepsis was its cause in Clark et al., 2001 [23] study.

Higher CTP score, increased INR values, more operative bleeding, higher blood, and plasma transfusion rates and lower mean platelet count were correlated with our patient mortality. In similar, increased CTP score, INR and the number of intra-operative blood and platelet transfusions were its predictors in Quillin III et al., 2013 [3] study.

Finally, It is clear in our series that high CTP score was predictor of all conversions to open surgery, 30-day morbidities and mortalities, moreover, 100% of CTP class C, and 62.5% of CTP class late B had morbidities, however, all our mortalities occurred in CTP class C (4 cases) and CTP class late B (1 case), so before performing LC in those higher scores, it is mandatory to do upgrading of liver condition to avoid those worse outcomes, but if upgrading fail, it is advisable to do the less invasive procedure (PC), however, if PC fail, it is fundamental to do the surgery only in complicated cholecystitis after failure of aggressive conservative treatment and after following the previously mentioned pre- and intraoperative precautions. In Conclusion, LC can be safely performed in cirrhotic patients. However, higher CTP and MELD scores, operative bleeding, more blood and plasma transfusion units, longer operative time, lower platelet count, and higher INR values are predictors of poor outcome that can be improved by proper patient selection and meticulous peri-operative care and by using Harmonic scalpel shears.

## Ethical approval

The approval by National liver institute (IRB), Menoufia university.

## Sources of funding

No source of funding for this research.

## Author contribution

Emad Hamdy Gad: Study design, data collection, writing, statistical analysis and publication.

Yasmin Kamel: data collection, writing, statistical analysis.

Ayman Alsebaey: data collection, writing.

Anwar Mohammed: data collection, writing.

Mohammed Al-sayed Abd-elsamee: data collection, writing, reference update.

## Research registration unique identifying number

Researchregistry4590.

## Guarantor

All the authors of this paper accept full responsibility for the work and/or the conduct of the study, had access to the data, and controlled the decision to publish.

## Provenance and peer review

Not commissioned, externally peer reviewed.

## Funding

No received grant or other financial support for this study.

## Declaration of competing interest

No conflict of interest to declare.
